# Dietary Vitamin B1 Intake Influences Gut Microbial Community and the Consequent Production of Short-Chain Fatty Acids

**DOI:** 10.3390/nu14102078

**Published:** 2022-05-16

**Authors:** Jonguk Park, Koji Hosomi, Hitoshi Kawashima, Yi-An Chen, Attayeb Mohsen, Harumi Ohno, Kana Konishi, Kumpei Tanisawa, Masako Kifushi, Masato Kogawa, Haruko Takeyama, Haruka Murakami, Tetsuya Kubota, Motohiko Miyachi, Jun Kunisawa, Kenji Mizuguchi

**Affiliations:** 1 Artificial Intelligence Center for Health and Biomedical Research, National Institutes of Biomedical Innovation, Health and Nutrition, 7-6-8 Saito-Asagi, Osaka 567-0085, Ibaraki, Japan; hkawashi@nibiohn.go.jp (H.K.); chenyian@nibiohn.go.jp (Y.-A.C.); attayeb@nibiohn.go.jp (A.M.); 2 Laboratory of Vaccine Materials, Center for Vaccine and Adjuvant Research and Laboratory of Gut Environmental System, National Institutes of Biomedical Innovation, Health and Nutrition, 7-6-8 Saito-Asagi, Osaka 567-0085, Ibaraki, Japan; hosomi@nibiohn.go.jp; 3 Department of Physical Activity Research, National Institutes of Biomedical Innovation, Health and Nutrition, 1-23-1 Toyama, Shinjuku 162-8636, Tokyo, Japan or ono-ha@kiryu-u.ac.jp (H.O.); konishi@toyo.jp (K.K.); tanisawa@waseda.jp (K.T.); haruka-m@fc.ritsumei.ac.jp (H.M.); miyachi@nibiohn.go.jp (M.M.); 4 Department of Nutrition, Kiryu University, 606-7 Azami, Kasakake-machi, Midori 379-2392, Gunma, Japan; 5 Faculty of Food and Nutritional Sciences, Toyo University, 1-1-1 Izumino, Itakura, Oura 374-0193, Gunma, Japan; 6 School of Sport Sciences, Waseda University, 2-579-15 Mikajima, Tokorozawa 359-1192, Saitama, Japan; 7 Department of Life Science and Medical Bioscience, Graduate School of Advanced Science and Engineering, Waseda University, 2-2 Wakamatsucho, Shinjuku 162-8480, Tokyo, Japan; mk.shuro@toki.waseda.jp (M.K.); haruko-takeyama@waseda.jp (H.T.); 8 Computational Bio Big-Data Open Innovation Laboratory, National Institute of Advanced Industrial Science and Technology, 3-4-1 Okubo, Shinjuku 169-8555, Tokyo, Japan; 9 Research Organization for Nano and Life Innovation, Waseda University, 513 Wasedatsurumaki, Shinjuku 162-0041, Tokyo, Japan; machacha0507@moegi.waseda.jp; 10 Institute for Advanced Research of Biosystem Dynamics, Waseda Research Institute for Science and Engineering, Graduate School of Advanced Science and Engineering, Waseda University, 3-4-1 Okubo, Shinjuku 169-8555, Tokyo, Japan; 11 Faculty of Sport and Health Science, Ritsumeikan University, 1-1-1 Nojihigashi, Kusatsu 525-0085, Shiga, Japan; 12 Department of Clinical Nutrition, National Institutes of Biomedical Innovation, Health and Nutrition, 1-23-1 Toyama, Shinjuku 162-8636, Tokyo, Japan; kubota@oha.toho-u.ac.jp; 13 Laboratory for Intestinal Ecosystem, RIKEN Center for Integrative Medical Sciences, 1-7-22 Suehiro, Tsurumi, Yokohama 230-0045, Kanagawa, Japan; 14 Division of Diabetes and Metabolism, The Institute for Medical Science, Asahi Life Foundation, 2-2-6 Nihonbashibakuro, Chuo 103-0002, Tokyo, Japan; 15 International Research and Development Center for Mucosal Vaccines, Institute of Medical Science, University of Tokyo, 4-6-1 Shirokanedai, Minato 108-8639, Tokyo, Japan; 16 Graduate School of Medicine, Graduate School of Pharmaceutical Sciences, Graduate School of Dentistry, Graduate School of Sciences, Osaka University, 1-1 Yamadaoka, Suita 565-0871, Osaka, Japan; 17 Department of Microbiology and Immunology, Graduate School of Medicine, Kobe University, 7-5-1 Kusunoki, Chuo, Kobe 650-0017, Hyogo, Japan; 18 Institute for Protein Research, Osaka University, 3-2 Yamadaoka, Suita 565-0871, Osaka, Japan

**Keywords:** 16S rRNA, gut microbiota, nutrients, SCFAs, butyrate, pathway, vitamins, thiamine, *Ruminococcaceae*, *Faecalibacterium*

## Abstract

The gut microbiota is closely related to good health; thus, there have been extensive efforts dedicated to improving health by controlling the gut microbial environment. Probiotics and prebiotics are being developed to support a healthier intestinal environment. However, much work remains to be performed to provide effective solutions to overcome individual differences in the gut microbial community. This study examined the importance of nutrients, other than dietary fiber, on the survival of gut bacteria in high-health-conscious populations. We found that vitamin B1, which is an essential nutrient for humans, had a significant effect on the survival and competition of bacteria in the symbiotic gut microbiota. In particular, sufficient dietary vitamin B1 intake affects the relative abundance of *Ruminococcaceae*, and these bacteria have proven to require dietary vitamin B1 because they lack the de novo vitamin B1 synthetic pathway. Moreover, we demonstrated that vitamin B1 is involved in the production of butyrate, along with the amount of acetate in the intestinal environment. We established the causality of possible associations and obtained mechanical insight, through in vivo murine experiments and in silico pathway analyses. These findings serve as a reference to support the development of methods to establish optimal intestinal environment conditions for healthy lifestyles.

## 1. Introduction

Currently, increasing efforts are being made globally toward supporting healthy lifestyles. Along with healthy eating and exercise habits, interest in a healthy intestinal environment has recently increased. The role of the intestinal environment on human health is well recognized and is an active research topic [1,2,3]. Disturbances in the intestinal environment lead to intestinal diseases and other adverse effects, such as allergies and diabetes mellitus [4,5]. The gut microbiota is one of the most influential factors in the intestinal environment. Indeed, the gut microbiome, where billions of microorganisms are clustered, forms a complex ecosystem, and dysbiosis or certain gut bacteria are directly or indirectly related to intestinal diseases [6], metabolic syndromes [7,8], and neurological disorders [9,10].

Gut bacteria are involved in the regulation of host health through the biosynthesis of several essential nutrients such as amino acids and vitamins, as well as biologically active components such as short-chain fatty acids (SCFAs). For example, butyrate, an SCFA produced by gut bacteria, plays a major role in maintaining health by regulating intestinal peristaltic movement, epithelial barrier function, and the immune system [11,12,13]. These functions exert protective effects against several diseases such as colorectal cancer, inflammatory bowel disease, graft-versus-host disease, diabetes, and obesity [14]. In addition to SCFAs, total gut microbial-synthesized nutrients, such as vitamins, are beneficial for both microbial and host metabolism. *Enterobacter agglomerans*, *Serratia marcescens*, and *Enterococcus faecium* produce vitamin K [15] and contribute to lowering the risk of cardiovascular diseases such as arteriosclerosis and coronary disease [16]. Exclusively synthesized by *Bacteroides fragilis* and *Prevotella copri*, vitamins B5 and B12 act as coenzymes in a wide range of host biochemical processes, including the production of acetylcholine and cortisol required for the normal function of the nervous system, and are associated with several disorders [17,18,19]. Thus, gut microbial composition and metabolite profile are indicative of an intestinal environment that maintains host health.

Diet also plays a critical role in the intestinal environment because it determines the microbial composition and amounts of metabolites produced, which influence the interaction between the intestinal environment and the host [20]. A twin study showed that environmental factors related to diet, drugs, and anthropometry have a greater determinant effect on the gut microbial community than heritable components [21]. In the framework of microbiome-related nutrition, the role of dietary fiber, which affects microbial composition, has been noted. Indeed, the intake amount and type of dietary fiber have been reported to affect gut microbial composition and SCFA production in mouse experiments and human cohort studies [22,23]. In addition, other dietary materials, including resistant starches, undigested proteins, fats, and plant chemicals, can reach the colon [24] and influence the gut microbial environment. Indeed, the effects of diet on the intestinal environment, composed of microbial communities and their metabolites, are complex, and findings from one clinical study can be difficult to reproduce in other studies [25]. This is presumably because each individual has a highly personalized gut microbial environment determined by their dietary history [26]. For these reasons, in order to properly evaluate the effect of human intestinal bacteria, it is necessary to study cohort units to understand specificity, and furthermore, a more objective evaluation is possible through verification by in vivo murine experiments or in silico.

In this study, we conducted a large-scale study of a healthy Japanese population to clarify the effects of nutrient intake on the intestinal environment. To establish the causality of possible associations and to gain mechanistic insights, we performed in vivo murine experiments and in silico pathway analyses. The findings of this study will provide insight into the control of gut bacteria and can contribute to the improvement of the intestinal environment.

## 2. Materials and Methods

### 2.1. Participants and Fecal Sample Collection

In this cross-sectional study, human fecal samples were collected from 257 healthy adult volunteers (age, 27–80 years; male, 65; female, 192) living in Tokyo, Japan [27]. The volunteers lived a healthy lifestyle and were followed up regularly at our institute. None of the participants included in the study had any history of cancer, cardiovascular disease, liver disease, or gastrointestinal disease, and volunteers who took antibiotics, laxatives, and antiflatulents within a month were excluded. Dietary habits were assessed using a brief-type self-administered diet history questionnaire (BDHQ) [28], and the amount of nutrient intake was calculated using dietary habit information (Table S1). The BDHQ is a four-page, fixed-portion questionnaire that asks about the consumption frequency of the selected food items to estimate the dietary intake of 58 food and beverage items in the preceding month. Informed consent was obtained from all participants. This study was approved by the Ethical Committee of the National Institutes of Biomedical Innovation, Health and Nutrition (Approval No. KENEI3-05).

Fecal samples were collected from 257 individuals in guanidine thiocyanate solution (TechnoSuruga Laboratory, Shizuoka, Japan) and then stored at 4 °C until DNA extraction for 16S rRNA gene amplicon sequencing. Another set of fecal samples was stored at −80 °C for SCFA measurements.

### 2.2. Murine Experiments

Female C57BL/6 mice (seven weeks old) were purchased from CLEA Japan (Tokyo, Japan) and used for experiments after pre-breeding for one week. The comparative control experiment was conducted by dividing it into a vitamin B1-deficient group (*n* = 4) and control group (*n* = 4). Vitamin B1-deficient and control diets with chemically defined components were purchased from Oriental Yeast (Tokyo, Japan), as previously described [29]. All experiments were approved by the Animal Care and Use Committee of the National Institutes of Biomedical Innovation, Health, and Nutrition (Approval No. DS27-48R10) and conducted in accordance with their guidelines. Mouse fecal samples were collected and stored at −80 °C until SCFA measurement and 16S rRNA gene amplicon sequencing.

### 2.3. SCFA Measurement

Human feces (5–10 mg) were mixed with 90 µL of MilliQ and 10 µL of 2 mM internal standard containing acetic acid, butyric acid, and crotonic acid for 5 min. The mixture was homogenized with 50 µL of HCl and 200 µL of diethyl ether and centrifuged at 3000 rpm for 10 min at room temperature. Next, 80 µL of the supernatant organic layer was transferred to a new glass vial and combined with 16 µL of *N*-*tert*-butyldimethylsilyl-*N*-methyltrifluoroacetamide as a derivatization reagent. The vials were immediately capped tightly with an electronic crimper (Agilent, Santa Clara, CA, USA), incubated for 20 min in an 80 °C water bath, and then left at room temperature in the dark for 48 h for derivatization. The derivatized samples were analyzed using a GC-MS-TQ8040 gas chromatograph mass spectrometer (Shimadzu, Kyoto, Japan). The injection was performed using an AOC-20i auto injector (Shimadzu, Kyoto, Japan). The capillary column was a BPX5 column (0.25 mm × 30 m × 0.25 µm; Trajan Scientific and Medical, Melbourne, Australia). Pure helium gas was used as the carrier gas and delivered at a flow rate of 1.2 mL min^−1^. The head pressure was 72.8 kPa with split (split ratio 30:1). The injection port and interface temperatures were 230 °C and 260 °C, respectively. This analysis measured 10 types of fecal SCFAs (C1:0-C6:0).

The mouse fecal samples were mixed with methanol at 100 mg/mL. After homogenization at 6500 rpm for 15 s two times (at 5 s intervals) using a Precellys 24 (Bertin Instruments, Montigny-le-Bretonneux, France), the mixture was centrifuged at 1600× *g* for 10 min at 4 °C. The supernatant was labeled with 2-nitrophenylhydrazide using an SCFA analysis kit (YMC, Kyoto, Japan), and the labeled samples were analyzed by high-performance liquid chromatography (HPLC) using an Ultimate 3000 (Thermo Fisher Scientific, Waltham, MA, USA) with a YMC-Pack FA column (YMC, Kyoto, Japan) according to the manufacturer’s instructions.

### 2.4. DNA Extraction and 16S rRNA Gene Amplicon Sequencing

The fecal sample mixture was mechanically disrupted using the bead-beating method. DNA was extracted using Gene Prep Star PI-80X (Kurabo Industries, Osaka, Japan). After DNA extraction, the V3-V4 region of the 16S rRNA gene was amplified using the following primers: forward, 5′-TCG GCA GCG TCA GAT GTG TAT AAG CGA CAG CCT ACG GGN GGC WGC AG-3′; reverse, 5′-GTC TCG TGG GCT CGG AGA TGT GTA TAA GAG ACA GGA CTA CHV GGG TAT CTA ATC C-3′ [30]. Amplicons were sequenced via the paired-end method using MiSeq (Illumina, San Diego, CA, USA). The overall procedure from fecal sampling to 16S rRNA sequencing was performed according to a previously described protocol [31].

### 2.5. Bioinformatics Analysis

The paired-end output from MiSeq was trimmed and merged before operational taxonomic units (OTUs) were picked. OTU classification and diversity analysis were performed using the QIIME pipeline (v. 1.9.1) [32]. All steps from trimming to diversity analysis were automatically performed according to previously described methods [33]. The OTUs were clustered against the SILVA 128 reference database [34] at 97% similarity using the USEARCH algorithm [35]. Taxonomic classification was performed using the SILVA 128 reference database from the phylum to the genus level.

### 2.6. Statistical Analysis

The output of the QIIME pipeline in the Biom table format was imported and analyzed using R (version 3.5.1). The Shannon diversity index was calculated using the *estimate_richness* function in the “phyloseq” R package. The beta diversity index, calculated by the Bray–Curtis distance using genus-level data, was generated using the *vegdist* function in the “vegan” R package. For enterotype analysis, we used the Jensen–Shannon distance, as previously described [36]. Principal coordinate analysis was conducted using the *dudi.pco* function in the “ade4” R package. Covariates of gut microbiome beta diversity variation were identified by calculating the association between continuous or categorical phenotypes and the coordinates of genus-level communities with the *envfit* function in the “vegan” R package. For correlation analysis, the dominant bacteria from the phylum to the genus level were defined as those with an average bacterial composition of at least 1%. We used the Wilcoxon rank sum test (*wilcox.test* function in “stats” R package) and Spearman correlation analysis (*cor* function in “stats” R package) for comparison and correlation analysis, respectively. Statistical analyses were performed using R (version 3.5.1). All statistical tests were two-sided, with a significance level of *p* < 0.05. Heatmaps were created using the “superheat” R package, and the other graphs were created using the R package “ggplot2”.

### 2.7. KEGG Pathway Analysis for Thiamine Synthesis

The presence or absence of enzymes involved in vitamin B1 synthesis was investigated using the complete genome sequences of bacteria belonging to the *Ruminococcaceae* (currently, *Oscillospiraceae*) and *Bacteroidaceae* families. From the bacteria registered in KEGG [37], we identified six species (eight organisms) of *Ruminococcaceae* and seven species (eight organisms) of *Bacteroidaceae* in our samples (Supplementary Table S1). First, we checked the presence of enzyme-coding genes essential for thiamine synthesis in the KEGG pathway maps (map00730) of these organisms. We then constructed a BLAST+ database [38] using the complete genome sequences of these organisms. Using the amino acid sequence of each gene of the thiamine synthesis pathway for these 16 organisms as query, a TBLASTN search was conducted on the BLAST database created. We obtained the gene names and their locations in their genomes from the TBLASTN search results. This method allows the identification of homologous genes, orthologs, paralogs, and genes that are not displayed to be present in the KEGG pathway. The amino acid sequence of the newly identified homolog gene was used for the TBLASTN search, and this process was repeated until new homologs were no longer found. The amino acid sequences of the identified genes were then classified using the Clustal Omega clustering and alignment tool [39].

## 3. Results

### 3.1. Ruminococcaceae Abundance Is Correlated with Vitamin B1 Intake in Humans

**Figure 1:**
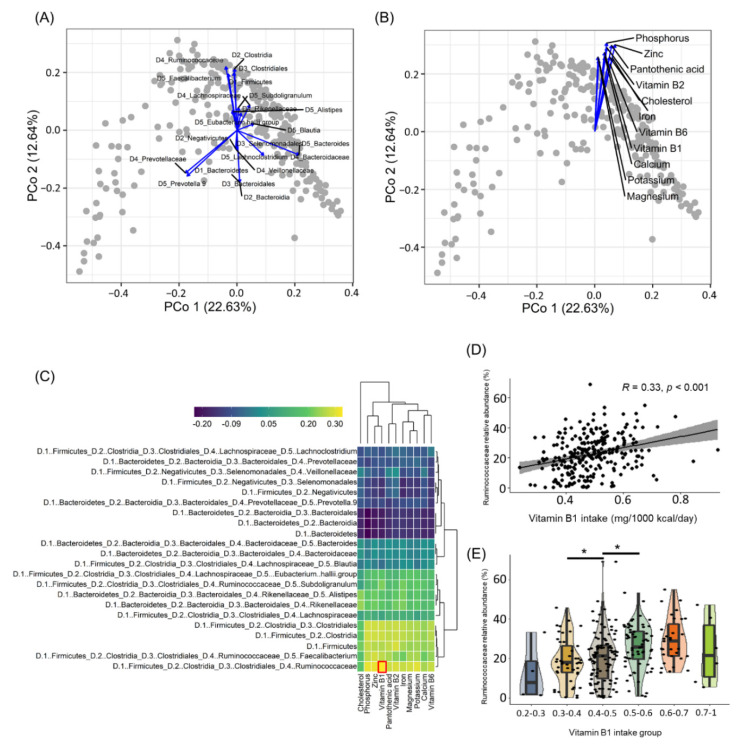
Correlation between gut bacterial community and nutrients. (**A**,**B**) Principal coordinate analysis of the Bray–Curtis distance for genus-level relative abundance data. Covariates of the bacterial relative abundance and nutrients intake were identified by calculating the association between each continuous data and genus-level community ordination with the *envfit* function in the vegan R package. Variables that have been significantly related to coordinates are described (*p*-value < 0.001). (**C**) Spearman correlation analysis was performed to elucidate the relationship between gut bacterial relative abundance and nutrients (*n* = 257; *p*-value < 0.05 [|r| > 0.12]). The red square is the result of the highest correlation. (**D**) Correlation between the *Ruminococcaceae* family and vitamin B1 is shown by scatterplots. (**E**) Boxplot for comparison of *Ruminococcaceae* family relative abundance between groups grouped by vitamin B1 intake (vitamin B1 intake groups; blue: 0.2–0.3 mg/1000 kcal/day, yellow: 0.3–0.4 mg/1000 kcal/day, brown: 0.4–0.5 mg/1000 kcal/day, dark green: 0.5–0.6 mg/1000 kcal/day, orange: 0.6–0.7 mg/1000 kcal/day, green: 0.7–1 mg/1000 kcal/day). * *p*-value < 0.05.

To clarify the effect of nutrient intake on gut bacteria, the correlations between intestinal bacterial community data obtained through 16S rRNA amplicon sequencing and nutrient intake data estimated from dietary intake history were confirmed in a cross-sectional study of 257 healthy Japanese subjects (Table S1). Principal coordinate analysis (PCoA)-envfit analysis allowed us to identify several broad classes of subjects (known as enterotypes), characterized by the abundance of specific bacterial genera, such as *Bacteroides*, *Faecalibacterium*, and *Prevotella* (Figure 1A). We next performed PCoA-envfit analysis to elucidate the relationship between the gut bacterial community and nutrient intake. We found that among several nutrients, B vitamins and micronutrients such as zinc and magnesium were related in a similar direction (Figure 1B), suggesting that these nutrients affect the survival of specific bacteria. To test this hypothesis, we examined the correlations between the bacteria and nutrients. As shown in the heatmap (Figure 1C), among bacteria with a similar orientation in the PCoA-envfit analysis (Figure 1A), the highest correlation was noted between the *Ruminococcaceae* family and vitamin B1 intake. In the scatterplot (Figure 1D), the distribution of variables for the correlation between *Ruminococcaceae* and vitamin B1 intake confirmed an unbiased positive correlation. Further, to confirm the effects of vitamin B1 intake, a comparative analysis by grouping vitamin B1 intake by 0.1 mg confirmed that the abundance of *Ruminococcaceae* in the gut was positively correlated with vitamin B1 intake up to 0.6 mg/1000 kcal/day (Figure 1E).

### 3.2. Vitamin B1 Intake Causatively Affects Gut Bacteria in Mice

**Figure 2:**
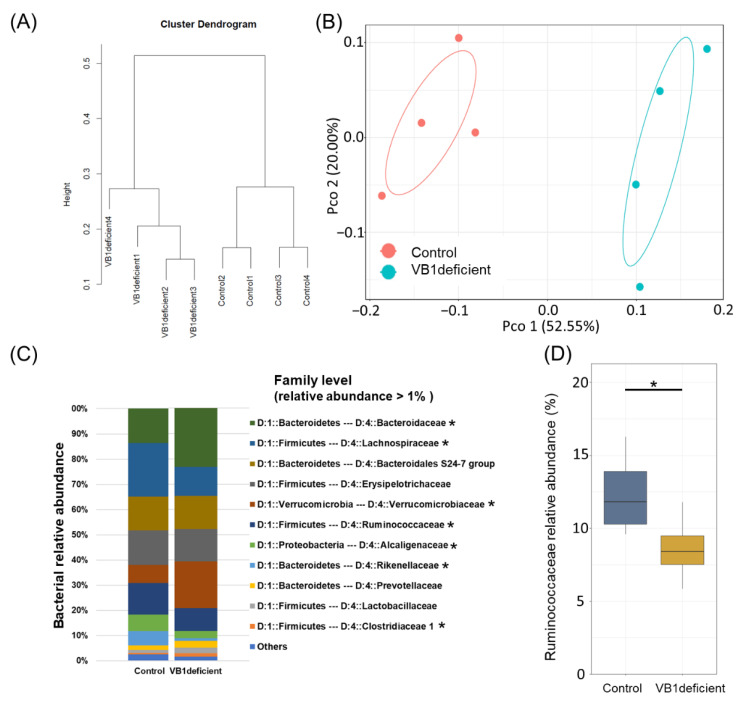
Correlation between gut bacterial community and vitamins in mouse data. (**A**) Cluster dendrogram generated using the Ward d2 algorithm for genus-level relative abundance. (**B**) Principal coordinate analysis results of the Bray–Curtis distance for genus-level relative abundance data (red, Control group; blue, Vitamin B1-deficient group). (**C**) Bar chart of mouse gut bacterial community composition at the family level. (**D**) Boxplot for comparison analysis of two groups in the *Ruminococcaceae* family. * *p*-value < 0.05.

Considering that vitamin B1 is an essential coenzyme for metabolic reactions in some bacteria, we conducted murine experiments to investigate whether vitamin B1 intake has a causative effect on gut bacteria. In this experiment, mice were fed with either a vitamin B1-sufficient or vitamin B1-deficient diet for one week, and the bacterial communities in their feces were examined using hierarchical clustering (Ward-d2 algorithm) and PCoA-envfit analysis (Figure 2A,B). The results revealed that vitamin B1 deficiency affected the mouse gut bacterial composition. Indeed, the gut bacterial community structures of the group fed a vitamin B1-deficient diet and the control group were significantly different. In particular, the vitamin B1-deficient group showed a decreased relative abundance of *Ruminococcaceae*, *Lachnospiraceae*, *Rhodospirillaceae*, and *Alcaligenaceae*, as well as an increased relative abundance of *Bacteroidaceae*, *Verrucomicrobiaceae, Christensenellaceae*, and *Clostridiaceae* (Figure 2C). Notably, despite the different structures of the gut bacterial community in humans and mice, the abundance of *Ruminococcaceae* was decreased in mice fed a vitamin B1-deficient diet (Figure 2D).

### 3.3. Ruminococcaceae Genomes Lack the Vitamin B1 Synthesis Pathway

**Figure 3:**
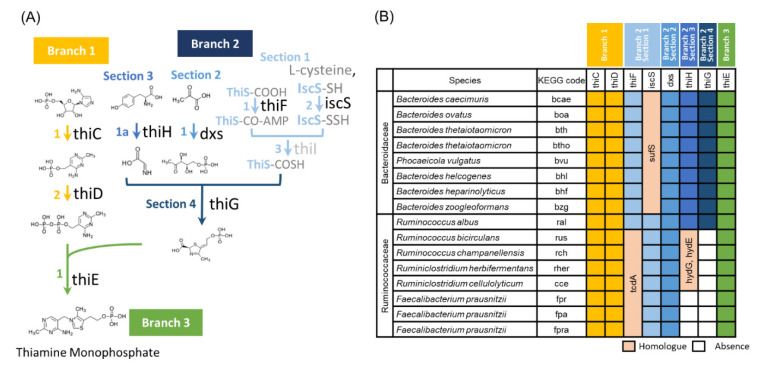
Pathway information and retention ratio for thiamin (vitamin B1) synthesis. (**A**) Pathways involved in vitamin B1 synthesis. (**B**) Retention ratio of specific enzymes in bacteria belonging to the *Ruminococcaceae* and *Bacteroidaceae* families.

We next investigated the presence or absence of enzymes involved in vitamin B1 synthesis in the complete genome sequences of bacteria belonging to the *Ruminococcaceae* (currently, *Oscillospiraceae*). The *Bacteroidaceae* was used as the control bacteria group, which is known as most domain bacteria in the gut. Our results did not show a correlation with dietic vitamin B1 in human cohort data, and the relative abundance was increased by vitamin B1 deficiency in murine experiments. Many bacteria in the gut can synthesize vitamin B1 on their own, whereas others cannot [40]. Therefore, it is possible that the difference in the effect of vitamin B1 deficiency between *Ruminococcaceae* and *Bacteroidaceae* is related to their ability to synthesize vitamin B1. To test this possibility, whole-genome data of bacteria belonging to these families were analyzed to investigate the presence or absence of genes encoding the enzymes necessary for vitamin B1 synthesis [41] (Figure 3A,B and Figure S2). *thiC*, *thiD*, *dxs*, and *thiE* were present in all bacteria of both *Bacteroidaceae* and *Ruminococcaceae*. The *iscS* was found in all *Ruminococcaceae* but not in *Bacteroidaceae*. *iscS* encodes cysteine desulfurase, which catalyzes the transfer of sulfur atoms from cysteine to the sulfur carrier protein *thiS*. *iscS* has many homologous genes, such as *nifS*, *csdA*, and *sufS*, of which sufS is present in all *Bacteroidaceae* and may replace *iscS* in this reaction. *Bacteroidaceae* contains all the other genes required for vitamin B1 synthesis, such as *thiF*, *thiG*, and *thiH*. The *thiI* encodes an enzyme involved in thiolidine formation that participates in sulfur transfer chemistry, but it has been reported to be nonessential [42]. These observations strongly suggest that *Bacteroidaceae* bacteria can synthesize vitamin B1 by themselves. On the other hand, *thiF*, *thiG*, and *thiH* were not identified in *Ruminococcaceae*, except in *Ruminococcus albus*. We found that *tcdA*, a paralog of *thiF*, exists in all *Ruminococcaceae* and that *hydE* and *hydG*, which are paralogs of *thiH*, exist in *Ruminococcaceae*, except in three *F. prausnitzii* strains. The proteins encoded by these genes may participate in the reaction in place of the *thiF* or *thiH*. However, there are no *thiG* or their paralogs in *Ruminococcaceae*, except in *R. albus*. The *thiG* encodes a thiazole synthase, which is essential for constructing the thiazole skeleton. Therefore, we can conclude that no *Ruminococcaceae* can synthesize vitamin B1, except for *R. albus*. Instead, these bacteria carry the *thiT*, which encodes a thiamine transporter, and thus can take up vitamin B1 from the environment. These results suggest that vitamin B1 intake affects the survival of *Ruminococcaceae* in the intestinal environment.

### 3.4. Vitamin B1 Intake and Acetate Production Are Correlated with Butyrate Production

**Figure 4:**
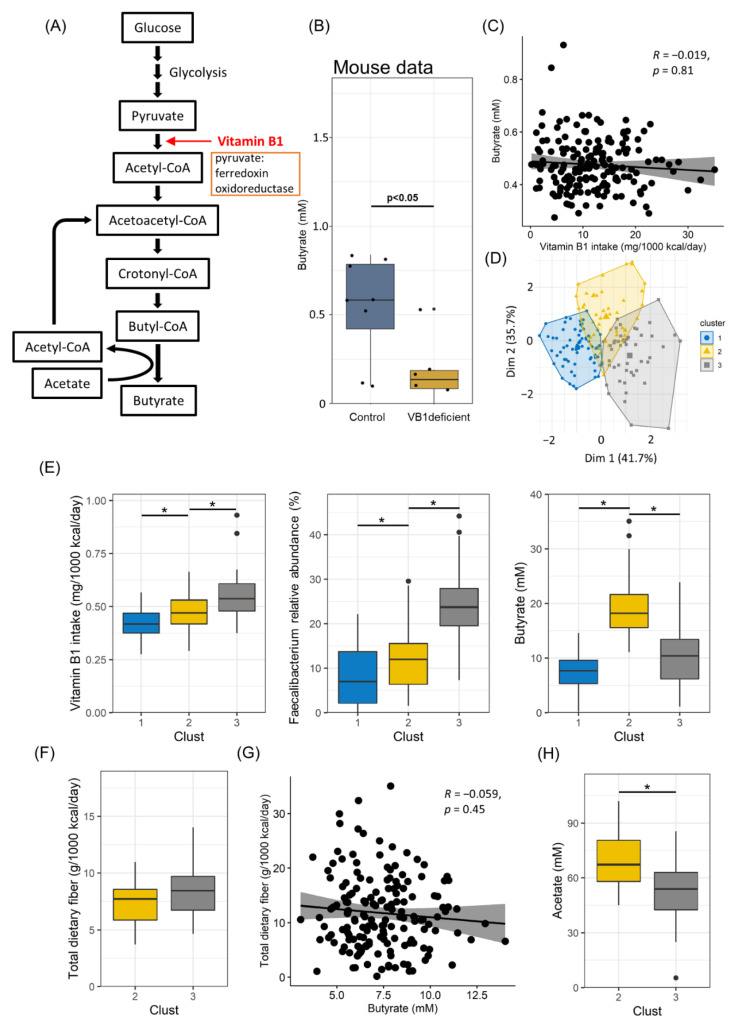
Effects of dietary fiber and gut bacteria on butyrate production. (**A**) Pathways for butyrate synthesis in *Faecalibacterium*. (**B**) Boxplot for comparison between the vitamin B1-deficient and control groups in terms of the amount of butyrate in mouse fecal samples. (**C**) Correlation between butyrate and vitamin B1 intake is shown by scatterplots. (**D**) HCPC analysis of vitamin B1 yield, butyrate amount in feces, and relative abundance of *Faecalibacterium*. (**E**) Boxplot for vitamin B1 intake (left), *Faecalibacterium* relative abundance (center), and butyrate amount in fecal samples (right), clustered by HCPC analysis. (**F**) Boxplot for comparison of two clusters in terms of total dietary fiber intake. (**G**) Correlation between total dietary fiber intake and vitamin B1 intake is shown by scatterplots. (**H**) Boxplot of comparison of two clusters in terms of acetate amount in fecal samples. * *p*-value < 0.05.

Bacteria belonging to *Ruminococcaceae* are known to synthesize butyrate. We found that *Ruminococcaceae* required vitamin B1 for their survival. These findings suggest that vitamin B1 intake affects butyrate production. This hypothesis is supported by the observation that *Faecalibacterium*, the most abundant genus in the *Ruminococcaceae* family, expresses pyruvate ferredoxin oxidoreductase, which requires vitamin B1 as a coenzyme for the conversion of pyruvate to acetyl-CoA in the butyrate production pathway [43] (Figure 4A and Figure S2). Consistent with these results, butyrate levels were significantly decreased in vitamin B1-deficient mice than in the control group (Figure 4B).

These findings prompted us to reanalyze the human data; however, no correlation between the butyrate amount in feces and vitamin B1 intake was noted (Figure 4C). Thus, we sought other factors to explain the absence of a correlation. We first performed Hierarchical Clustering on Principal Components (HCPC) analysis and identified three clusters (Figure 4D). A comparative analysis using boxplots revealed that butyrate production was associated with the amounts of vitamin B1 and *Faecalibacterium* in clusters 1 and 2. Low and high levels of vitamin B1, *Faecalibacterium*, and butylate were observed in clusters 1 and 2, respectively (Figure 4E). In contrast, cluster 3 showed different results, especially low butylate production even under a high intake of vitamin B1 and in the presence of *Faecalibacterium* (Figure 4E). We first assumed dietary fiber as a factor influencing butyrate production, as dietary fiber is a parent substrate for butyrate production. However, there was no difference in the amount of dietary fiber intake between clusters 2 and 3 (Figure 4F). In addition, no correlation was found between dietary fiber intake and fecal butyrate amount (Figure 4G), although some bacteria, such as *Faecalibacterium*, were positively correlated with butyrate and propionate (Figure S2). We then considered the effect of acetate, which is a SCFA and an important substrate for butyrate production. We found that the amount of fecal acetate in cluster 3 was lower than that in cluster 2 (Figure 4H). These findings collectively suggest that the vitamin B1-mediated maintenance of *Faecalibacterium* and acetate is a critical factor determining the amount of butyrate in the gut.

## 4. Discussion

In this study, we revealed the effect of dietary vitamin B1 intake on the intestinal environment in a human cohort study and verified the results in murine experiments and pathway analysis. We highlight the following findings to understand the relationship between the gut microbiome and dietary vitamin B1 intake: (a) sufficient dietary vitamin B1 intake can increases the relative abundance of *Ruminococcaceae*; (b) these bacteria require dietary vitamin B1 because they lack the de novo vitamin B1 synthetic pathway; (c) dietary vitamin B1 is essential for the production of butyrate mediated by these butyrate-producing bacteria, but their butyrate production capacity can be affected by the amount of acetate in the intestinal environment.

Vitamin B1 is important as a cofactor of several enzymes, such as α-ketoglutarate dehydrogenase and pyruvate dehydrogenase [40,44]. Many bacteria, including gut bacteria, require vitamin B1 for their metabolic activity, such as energy generation for their growth [18]. The sources of vitamin B1 are both diet and gut bacteria. Because some bacterial species can synthesize vitamin B1, in situations where the supply of dietary vitamin B1 is restricted, the abundance of certain bacteria decreases [41]. Indeed, several gut bacteria express vitamin B1 transporters to obtain extracellular environmental vitamin B1 [18]. A metagenomic analysis of human gut bacteria reported that specific bacteria, such as Bacteroidetes and Actinobacteria, can produce vitamin B1, but most members of Firmicutes are unable to produce vitamin B1 [40]. Consistent with the previous report, our pathway analysis revealed that *Ruminococcaceae* (belonging to the Firmicutes phylum) lacked the vitamin B1 synthetic pathway. Thus, the *Ruminococcaceae* family requires a vitamin B1 supply from the extracellular environment, such as the host diet, for their coexistence in the fiercely competitive intestinal environment.

In addition, vitamin B1 is involved in butyrate synthesis as a cofactor. Our mouse experiments clearly showed that fecal butyrate levels were significantly decreased under vitamin B1-deficient conditions, showing that dietary vitamin B1 affects intestinal butyrate levels in addition to gut microbial composition. In contrast, our human data did not show a direct correlation between vitamin B1 intake and fecal butyrate amount. It is generally difficult to establish a clear correlation owing to individual differences in the absorption and use of nutrients in the case of human cohort data, unlike in mouse experiments. Despite this, we assumed that intestinal acetate would explain the discrepancy. Acetate has two major metabolic roles in butyrate synthesis. The first is to remove CoA from butyl-CoA, and the second is as a butyrate intermediate product, as CoA is obtained from butyl-CoA and converted into acetyl-CoA (Figure 4A). Therefore, acetate is thought to have a significant effect on butyrate synthesis. Considering these metabolic roles, the possibility of butyl-CoA accumulation can be considered if the butyl-CoA amount is insufficient in the intestine despite the abundance of dietary fiber, vitamin B1, and butyrate producers.

Interestingly, dietary fiber is thought to have a significant effect on butyrate production because it is a parent substrate for butyrate production. Nevertheless, in our human data, no correlation was found between dietary fiber intake and fecal amount of butyrate (Figure 4G). Dietary fiber intake is indeed important because it is the main food for intestinal bacteria. However, the higher the consumption of dietary fiber, the more unlikely it is to affect butyrate production. Despite many confounding factors, it has been shown that the gut bacteria affects the production of butyrate more than the substrate (Figure S3); thus, these results indicate that to increase butyrate production, it is necessary to prepare an environment where related bacteria can grow. In the mouse study, the fecal propionate amount also showed a decreasing trend in vitamin B1-deficient mice, but there was no significant difference, and the fecal acetate amount showed no effect at all. Therefore, vitamin B1 intake is thought to be specifically related to butyrate production (Figure S4). These findings collectively suggest that the vitamin B1-mediated maintenance of butyrate producers and acetate is a critical factor determining the amount of butyrate in the gut.

A meta-analysis of reports that examined the relationship between vitamin B1 intake and urinary vitamin B1 excretion showed that urinary vitamin B1 excretion increased in a dose-dependent manner, with a marked increase in urinary vitamin B1 excretion at an inflection point of vitamin B1 intake of 0.35 mg/1000 kcal (WHO Technical Report Series No. 362). Rats in excretion equilibrium discharge 25–35% of vitamin B1 through urine and 20–30% through feces [45]. Therefore, it is thought that a vitamin B1 intake of at least 0.35 mg/1000 kcal per day is required to supply vitamin B1 to the bacteria present in the large intestine. As the recommended dietary allowance of vitamin B1 is 0.49 mg/1000 kcal, it seems that the intake of dietary vitamin B1 at a similar level may affect gut bacteria. Our results suggested that a vitamin B1 intake up to 0.6 mg/1000 kcal per day could increase the abundance of *Ruminococcaceae* despite individual differences. Because vitamin B1 is a water-soluble vitamin, and risks posed by the excessive consumption of vitamin B1 have not been reported, it is recommended that a sufficient amount be consumed in daily life.

A limitation of the present human cohort study was that the influence of the source or recipe of nutrients was not considered, because nutrient intake was estimated based on the data of eating habits collected via a BDHQ. Second, because the results were obtained from observational studies, they might have been affected by bias. Those who participated in the study were interested in their health, and in fact, they had good overall nutritional intake, including vitamin B1. These limitations can be resolved by randomly setting more diverse data or conducting intervention studies on vitamin B1 intake using human cohorts. Furthermore, butyrate is well-known to play an important role in maintaining health through intestinal peristalsis, epithelial barrier function, and immune system regulation [11,12]; hence, evaluating the effectiveness of vitamin B1 intake in preventing or treating butyrate-associated diseases will be of great benefit.

In conclusion, the importance of gut bacteria in disease prevention and physical condition management has been recognized recently, and various related studies have been conducted. However, the judgment varies greatly among individuals, and hence it is still difficult to obtain clarity on this matter. In addition, probiotics and prebiotics have been developed to maintain a healthy intestinal environment, but their effects differ among individuals. In this study, we found that vitamin B1, which is an essential nutrient for humans, can have a significant effect on the survival and competition of bacteria in the symbiotic gut microbiota, and we further demonstrated that vitamin B1 can be involved in the production of butyrate, along with the amount of acetate in the intestinal environment. Therefore, the findings of this study are expected to lead to a new concept of healthy lifestyle that emphasizes on building an optimal intestinal environmental condition that enhances human health by modifying the intestinal microbial community through ingestion of appropriate dietary components.

## Data Availability

The human gut sequencing data from this study have been deposited in the DDBJ Sequence Read Archive under accession number DRA010837.

## Supplementary Materials

The following supporting information can be downloaded at: https://www.mdpi.com/article/10.3390/nu14102078/s1, Figure S1: Distribution of genera belonging to the *Ruminococcaceae* family in this study; Figure S2: Correlation analysis heatmap generated using Spearman analysis between SCFA and dietary fiber intake, as well as between SCFA and gut bacteria in human data; Figure S3: Linear regression analysis results for butyrate amount in human fecal samples; Figure S4: Boxplot for comparison between the vitamin B1-deficient and control groups in terms of acetate and propionate amounts in mouse fecal samples; Table S1: List of phenotype metadata used in this study; Table S2: Whole genome sequencing data and information used to investigate the vitamin B1 synthesis pathway.
