# Supplementary material for: Dietary Vitamin B1 Intake Influences Gut Microbial Community and the Consequent Production of Short-Chain Fatty Acids
Source: Nutrients. 2022 May 16;14(10):2078. doi: 10.3390/nu14102078 (PMC9147846; doi:10.3390/nu14102078)
Supplement: Supplementary file 1 [file nutrients-14-02078-s001.zip › nutrients-1696990-supplementary figures.pdf]

Supplementary Data

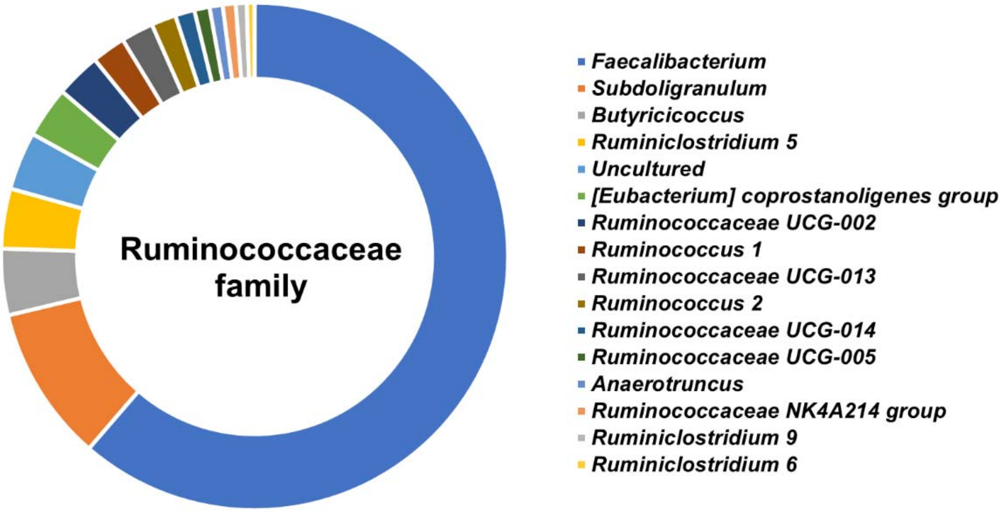

Figure S1. Distribution of genera belonging to the Ruminococcaceae family in this study.

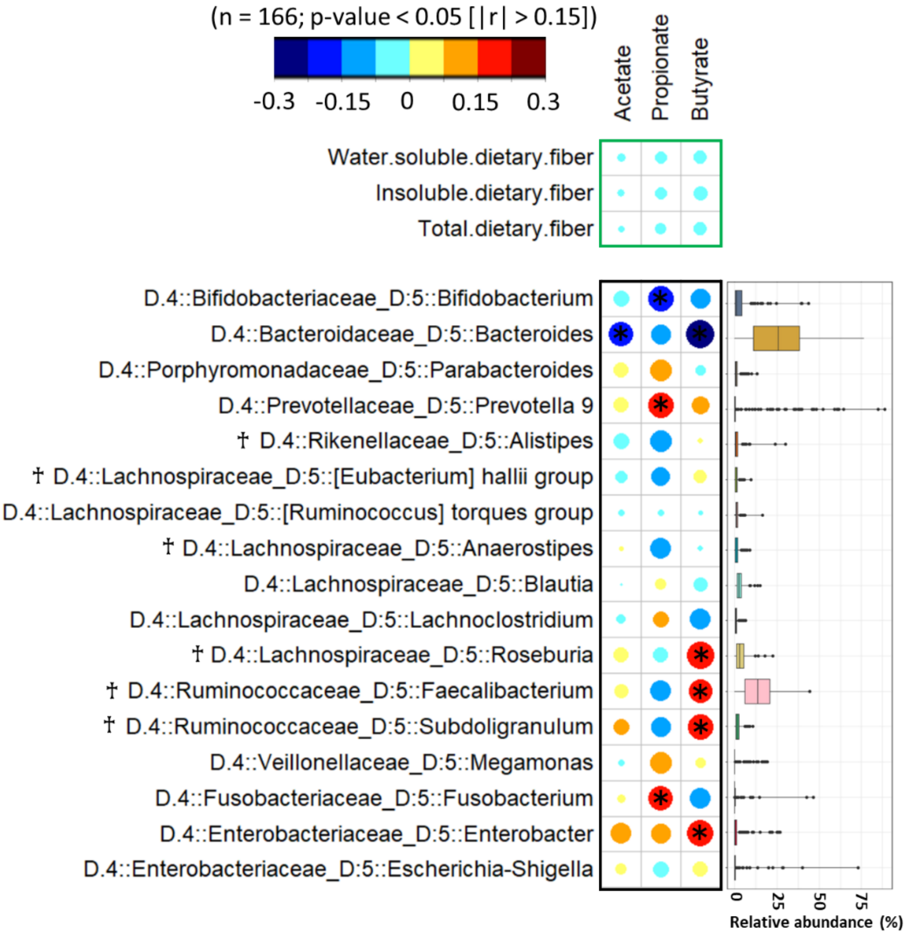

Figure S2. Correlation analysis heatmap generated using Spearman analysis between SCFA and dietary fiber intake as well as between SCFA and gut bacteria in human data († butyrate producer at the genus level, * p-value < 0.05).

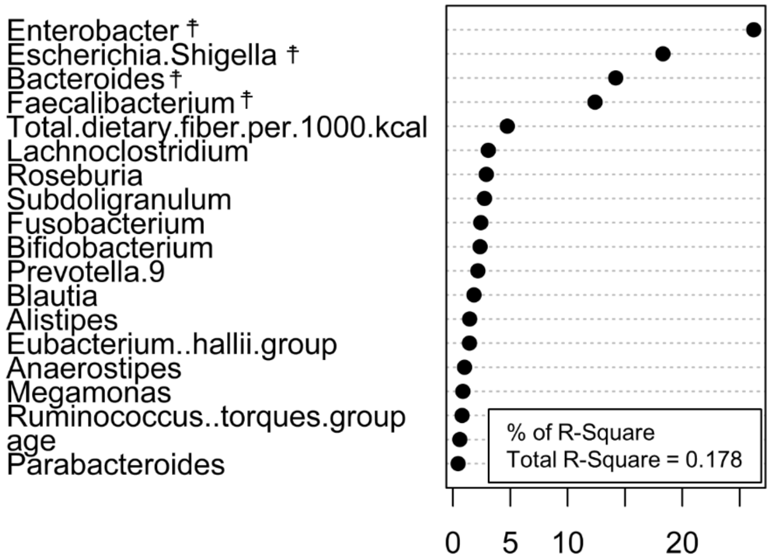

Figure S3. Linear regression analysis results for butyrate amount in human fecal samples. To identify non-redundant determinants of butyrate amount in human fecal samples, the covariates were sub-selected by forward stepwise redundancy analysis with the lm function and step function in the "stats" R package. † Remaining covariates after stepwise redundancy analysis (R-Square = 0.12).

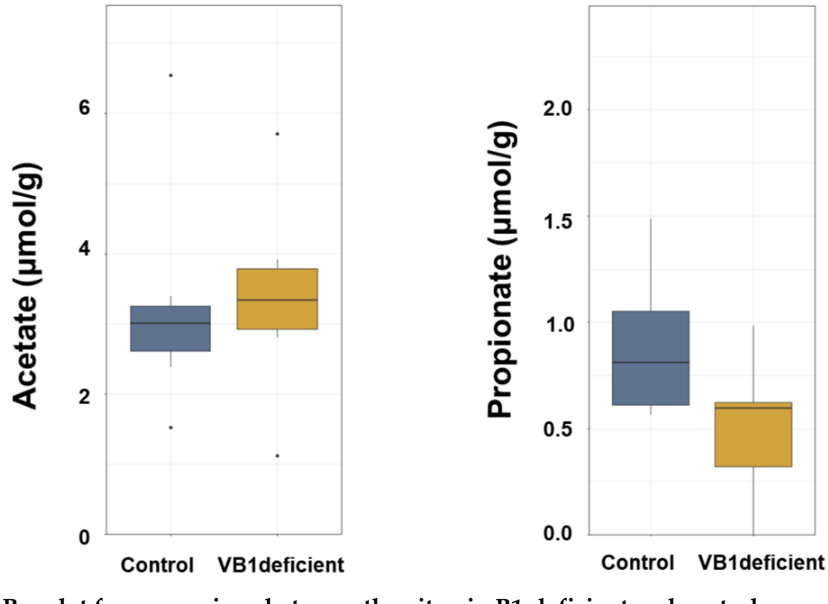

Figure S4. Boxplot for comparison between the vitamin B1-deficient and control groups in terms of acetate and propionate amounts in mouse fecal samples.
